# Use of Polyamidoamine Dendrimers in Brain Diseases

**DOI:** 10.3390/molecules23092238

**Published:** 2018-09-03

**Authors:** Maria Florendo, Alexander Figacz, Bhairavi Srinageshwar, Ajit Sharma, Douglas Swanson, Gary L. Dunbar, Julien Rossignol

**Affiliations:** 1 College of Medicine, Central Michigan University, Mt. Pleasant, MI 48859, USA; flore1mv@cmich.edu (M.F.); figac1am@cmich.edu (A.F.); srina1b@cmich.edu (B.S.); 2 Field Neurosciences Institute Laboratory for Restorative Neurology, Central Michigan University, Mt. Pleasant, MI 48859, USA; dunba1g@cmich.edu; 3 Program in Neuroscience, Central Michigan University, Mt. Pleasant, MI 48859, USA; 4 Department of Chemistry & Biochemistry, Central Michigan University, Mt. Pleasant, MI 48859, USA; swans1d@cmich.edu; 5 Department of Psychology, Central Michigan University, Mt. Pleasant, MI 48859, USA; 6 Field Neurosciences Institute, St. Mary’s of Michigan, Saginaw, MI 48604, USA

**Keywords:** PAMAM dendrimers, nanoparticles, blood-brain barrier, DNA delivery, drug delivery, neurodegenerative diseases

## Abstract

Polyamidoamine (PAMAM) dendrimers are one of the smallest and most precise nanomolecules available today, which have promising applications for the treatment of brain diseases. Each aspect of the dendrimer (core, size or generation, size of cavities, and surface functional groups) can be precisely modulated to yield a variety of nanocarriers for delivery of drugs and genes to brain cells in vitro or in vivo. Two of the most important criteria to consider when using PAMAM dendrimers for neuroscience applications is their safety profile and their potential to be prepared in a reproducible manner. Based on these criteria, features of PAMAM dendrimers are described to help the neuroscience researcher to judiciously choose the right type of dendrimer and the appropriate method for loading the drug to form a safe and effective delivery system to the brain.

## 1. Introduction

The major brain diseases that will be the focus of this review include neurodegenerative diseases and an aggressive form of primary brain cancer known as glioblastoma multiforme (GBM). Neurodegenerative diseases, such as Huntington’s disease (HD), Alzheimer’s disease (AD), or Parkinson’s disease (PD), are pathologies consisting of neuronal degeneration causing loss of function of the affected part of the brain. Since most types of neurons are unable to regenerate, the death of these cells results in an irreversible pathology with detrimental outcomes [1]. Disease presentations for neurodegenerative diseases vary from memory loss to lack of motor coordination, depending on the area of the brain that is involved. Despite the difference in clinical symptoms, all neurodegenerative diseases progress to the same outcome: irreversible cell death. Patients who suffer from neurodegenerative diseases must live with their debilitating effects, making developments for treatments increasingly important. The causes of neurodegenerative diseases are mostly unknown [2]. However, inflammation seems to be closely associated with these diseases. Under the broad category of different forms of glioma that arise from the glial cells, the most aggressive form of tumor is the GBM or grade-4 astrocytoma. This form of cancer does not have a cure and the patients diagnosed with glioblastoma have a high rate of mortality and morbidity. GBM is also highly associated with brain inflammation [3].

Many excellent reviews on the chemistry and biomedical applications of polyamidoamine (PAMAM) dendrimers are available [4,5,6,7,8,9]. PAMAM dendrimers are by themselves complex macromolecules and their appropriate use for a specific application can be confusing to many non-chemists. Excellent recent reviews on the use of dendrimers for the central nervous system are available [10].The intent of this review is not to summarize all of the work related to the applications of PAMAM dendrimers to brain diseases. Instead, we shall look at the physical and chemical features of PAMAM dendrimers that are beneficial for their potential uses in neuroscience research and eventually in the clinic, keeping two critical criteria in mind:

- Safety of the dendrimer formulation
- Reproducibility in preparing the dendrimer formulation

Safety is always an essential requirement for all drugs, which is obvious and deserves no further comments. However, reproducibility of dendrimer preparation is an important consideration for clinical use of macromolecular drugs or macromolecular drug excipients. Preparation of large [molecular weight (MW) > 1000 Da] synthetic macromolecules such as PAMAM dendrimers and other nanomolecules, unlike small molecule drugs (MW < 500–1000 Da), is a challenging task for the organic chemist. Besides the target compound, closely related chemical species are often formed due to side reactions and are difficult to remove by well-established purification methods. The final macromolecule formulation is typically plagued with large batch-to-batch variations that may not meet the stringent requirements for regulatory approvals.

## 2. Features of PAMAM Dendrimers Useful for Neuroscience Applications

Dendrimers are branched nanomolecules. There are many different types of dendrimers that have been synthesized with potential uses in the treatment of brain diseases. Examples include carbosilane dendrimers, polylysine dendrimers, phosphorus-containing dendrimers, and PAMAM dendrimers [11,12,13].

**Figure 1:**
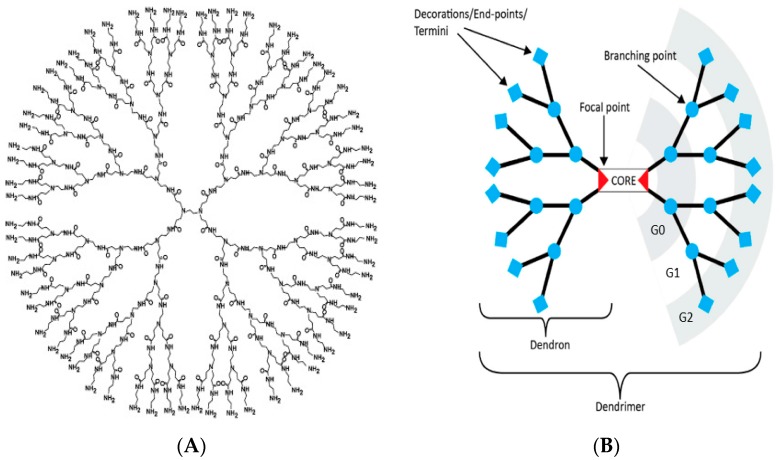
(**A**) A G4 polyamidoamine (PAMAM) dendrimer [37].(**B**) Every component of a dendrimer can be controlled including its core composition, size, shape, and surface (adapted from [38]).

PAMAM dendrimers are one of the earliest dendrimers synthesized and made commercially available [8]. As shown in Figure 1B, a PAMAM dendrimer consists of: (1) a core; (2) branches composed of amide groups emanating from a branching point (tertiary amine) that form the walls of cavities; and (3) terminal groups. The earliest core reported was ethylenediamine (EDA). Subsequently PAMAM dendrimers were synthesized from a variety of other cores such as diaminobutane (DAB), diaminohexane, diaminododecane and cystamine [14]. As with disulfide-containing proteins, the disulfide bonds in cystamine-core dendrimers may be cleaved by reducing agents such as glutathione and beta-mercaptoethanol to yield two half-dendrimers known as dendrons [15]. The branches of PAMAM dendrimers contain amide bonds that are similar to the peptide backbones of proteins. Dendrimers with termini composed of primary amines (−NH_2_), hydroxyls (−OH) or carboxyls (−COOH) were the earliest type of PAMAM dendrimers reported and commercially available. The amine-terminated PAMAM dendrimers became popular among biologists and biomedical researchers since they were commercially available and, more importantly, could easily undergo bio-conjugation reactions with other molecules, using protocols and reagents that were well-established for proteins. For example, these dendrimers are easily conjugated to fluorescent dyes using reactive fluorescent reagents made for protein-labeling such as fluorescein isothiocyanate (FITC) or NHS-Cyanine 5.5 (Cy5.5) for tracking purposes in biological systems.

Some of the important features of PAMAM dendrimers for neuroscience applications include:

- High stability
- High water-solubility
- Small size
- Precision
- Presence of cavities
- Surfaces that can be readily modified

Thermogravimetric analysis data shows that PAMAM dendrimers are stable to heat; they start degrading over 200 °C [16,17]. Proteins rely on weak interactions such as salt bridges, hydrogen bonds and hydrophobic forces for their shape. However, the three-dimensional shapes of PAMAM dendrimers are mostly due to their dendritic architecture with some contribution by weak forces. For this reason, PAMAM dendrimers may be stored frozen (dried or as solutions) and thawed for use without any significant denaturation or aggregation. The use and storage of protein drugs requires many precautions. However, this is not the case for PAMAM dendrimers if they become routinely used in drug formulations. This is especially important for use in neurodegenerative diseases due to the chronic treatment involved and patients that often have some degree of memory loss who may leave the drugs at room temperature instead of in the refrigerator.

Like non-membrane proteins, PAMAM dendrimers are highly water-soluble if their surface is composed of functional groups (e.g., −NH_2_, −OH, COOH) that hydrogen-bond with water. Aqueous solutions as high as 100–200 mg/mL dendrimer are readily formed. The water solubility of chemicals is an important factor that affects their absorption, distribution, metabolism and excretion (ADME). Nanomolecules with hydrophilic surfaces are less likely to become attracted to plasma proteins (opsonization) and thus less prone to be cleared from the bloodstream via engulfment by cells of the reticuloendothelial system. It is therefore important to keep in mind that attaching chemicals such as drugs and fluorescent dyes to the surface of dendrimers will decrease the solubility of the resulting nanomolecules and also affect their ADME. This is a serious practical problem since academic research requires attachment of fluorescent dyes or other imaging agents to understand the biological behavior of the nanomolecules. An example of the effect of dendrimer solubility on its biological properties was demonstrated by Dougherty and colleagues [18]. The reaction of attaching fluorescent dye to PAMAM dendrimers (or any macromolecules with numerous reactive sites) not only gives a Poisson distribution of dye:dendrimer ratios, but the cellular uptake increases with the number of dye molecules on the surface of the dendrimer. Since dyes are hydrophobic, their attachment on the hydrophilic dendrimer surface creates hydrophobic sites that interact with the plasma membrane and increase their cellular uptake via adsorptive endocytosis. Another example is evident in an elegant report by Albertazzi and co-workers. Using two-photon microscopy, they showed that after injection into the brain cortex of live animals, amine-terminated PAMAM G4 was able to diffuse from the injection site while the less water-soluble G4-C12 (similar dendrimer with 25% of surface coated with C12 chains) remained at the injection site. Clearly, good water solubility will be important for the dendrimer to distribute its drug payload throughout the brain. In addition, the G4-C12 PAMAM dendrimers were over three-fold more toxic to neurons than the G4 amine-terminated dendrimer. Due to its water solubility, the G4 dendrimer readily diffused in the brain of living animals and therefore did not form high local concentrations of the dendrimer to be toxic; neurons with the dendrimer were still shown to be physiologically active. However, the poor water solubility of the G4-C12 led to poor diffusion and high local concentration at the site of injection, resulting in neuronal death via apoptosis. Even glial cell activation was none to minimal after exposure to the G4 amine-terminated PAMAM dendrimer. This is an important concept to consider when trying to extrapolate cell culture data to in vivo conditions. Since concentration drives many biological processes, good water solubility diminishes the chances of highly localized concentrations of the nanomolecules. Thus, although G4 amine-terminated dendrimers may show toxicity under cell culture conditions (typically at micromolar concentrations),their effect in the dynamic and changing in vivo conditions (e.g., rapid dilution to nanomolar concentrations due to diffusion) might be surprisingly different [19].

Another critical feature of chemicals that affect their ADME is size. Commonly used PAMAM dendrimers such as G4, G5 and G6 have molecular weights in the range of approximately 14–56 kDa. These sizes are comparable to common proteins such as cytochrome c (MW ~12 kDa), lysozyme (MW ~14 kDa) and albumin (MW ~67 kDa). In terms of dimensions, the PAMAM dendrimers (G1–G10) range from about 1–15 nm. Compared to other nanomolecules such as liposomes and those made from polymers, PAMAM dendrimers are the smallest in size. Since the molecular weight cut-off (MWCO) for the glomerular filtration membrane in healthy kidneys is about 45,000 Da or 6–8 nm for nanoparticles [20,21], one can expect the commonly-used PAMAM dendrimers, especially G4 and G5, to be eventually excreted out of the body after administration. This is important to avoid potential accumulation and toxicity of the nanomolecules, especially with chronic use which is often the case for treatment of neurodegenerative diseases. However, this also means that the dendrimers have shorter half-lives when injected into the body. Larger generations >G6 or PEGylated dendrimers tend to stay longer in the bloodstream for distribution to the organs [22,23]. The size of the nanomolecule is also important for its ability to leave the bloodstream and enter the tissue. The distance between endothelial cells lining blood vessels varies considerably from as large as over 100 nm in liver capillaries to less than 1 nm in the blood brain barrier (BBB). This implies that it will be difficult for G4 or G5 PAMAM dendrimers (or other nanomolecules) to simply travel between endothelial cells of the BBB unless the barrier is compromised due to neuroinflammation [24]. Sarin and colleagues showed that dendrimers smaller than 11.7–11.9 nm were able to cross the compromised blood-brain barrier of rodents with malignant gliomas [25]. Nance and colleagues showed that neutral G4 PAMAM dendrimers composed of 100% −OH (G4-OH) are able to cross the compromised BBB and diffuse into the brain parenchyma to localize to activated glia of diseased mouse models [26]. In fact, this difference in size between the healthy and disrupted BBB barrier offers PAMAM dendrimers the advantage of traversing the compromised barrier compared to other nanomolecules such as liposomes, which are much larger.

Macromolecules made by nature such as proteins and nucleic acids are precise molecules with well-defined molecular weights. On the other hand, laboratory synthesis of macromolecules is challenging and often yields contaminating macromolecules with varying molecular weights besides the target compound; this is often due to side reactions and the difficulty in purifying closely related species. Reproducible synthesis of nanomolecules and good characterization data are critical for their successful use in humans. There must be minimal lot-to-lot variation between batches of dendrimers or other nanomolecules. The methods for PAMAM synthesis and characterization are well-established. By using excess reagents (such as ethylenediamine) during PAMAM dendrimer synthesis, the formation of side products (such as oligomers and looped surfaces) is dramatically reduced. As previously mentioned, it is important to note that although the parent dendrimers may be precise (low polydispersity), attachment of fluorescent dyes or other chemicals to the surface (carried out without using reagent excess) often yields a heterogenous mixture of products with varying ADME profiles. In addition, when administered into the body, such macromolecules may become altered as their surface groups become detached via metabolism, potentially resulting in the formation of toxic products. For example, a PAMAM dendrimer with 10 drugs attached to its surface via hydrolysable ester bonds is synthesized and tested for safety. After administration to an animal, the dendrimer inside the bloodstream or cells may be enzymatically degraded to yield a dendrimer with six drugs attached. The ADME or safety profile of this new product is unpredictable and may lead to long-term complications. Evidence for such a phenomenon is well-known among dendrimer researchers. For example, as mentioned earlier, even the attachment of a few fluorescent dye molecules to the surface of a dendrimer alters its cellular uptake [18].

The precision of PAMAM dendrimers and their derivatives is best appreciated by high-resolution separation techniques that have been well-established for precise macromolecules such as proteins. These techniques include reverse-phase high performance liquid chromatography (RP-HPLC) and electrophoresis [27,28]. Although non-separation techniques such as Nuclear magnetic resonance (NMR), infra-red (IR) or UV-visible spectrophotometry yield valuable characterization data, they do not show the purity of the nanomolecules. When analyzed by electrophoresis on polyacrylamide gels (typically at 1 mg/mL concentration), PAMAM dendrimers appear as discrete bands similar to proteins. Polymers, on the other hand, are seen as smears on these gels [29]. Although electrophoresis has been frequently used for the characterization of amine-surface dendrimers, the technique also works very well for separation of all types of PAMAM dendrimers (and their derivatives), irrespective of their cores or surface groups, since all PAMAM dendrimers have tertiary amines (at the branching points, Figure 1A) that are positively charged under acidic conditions. Charges are necessary for migration in electrophoresis gels. Electrophoresis has been a gold standard for determination of protein purity for several decades and is clinically an important tool for detection of various macromolecules in blood such as serum proteins, isozymes, hemoglobin variants and lipoproteins. Unfortunately, many reports on the synthesis and biomedical applications of PAMAM dendrimers do not show the purity of their parent or modified dendrimers by electrophoresis. For example, we have run a variety of PAMAM dendrimers by polyacrylamide gel electrophoresis (PAGE) and found that EDA-core PAMAM dendrimers are less pure than DAB-core dendrimers, yet many neuroscience researchers still use the former nanomolecules.

Another useful feature of PAMAM dendrimers for neuroscience applications is the presence of cavities. When placed in aqueous solutions, these cavities are hydrophobic and offer sites for loading small molecule drugs by a process known as encapsulation. Since the PAMAM dendrimer itself is extremely water-soluble, encapsulation within the cavities is an ideal way to solubilize poorly water-soluble drugs in aqueous solutions. For example, Igartúa and colleagues found that a G4 PAMAM dendrimer with an amine surface (or a G4.5 PAMAM dendrimer with a carboxyl surface) could encapsulate about 20 molecules of the anti-epileptic drug, carbamazepine, which has potential for treating neurodegenerative diseases [30]. Recent reviews on the use of PAMAM dendrimers for encapsulating drugs are available [5,31]. Encapsulation is also how cyclodextrins (cyclic sugar molecules popular in the drug industry) help to solubilize drugs. A cyclodextrin has one cavity per molecule while a G4 PAMAM dendrimer has several cavities per molecule [32]. Many drugs, including those used for treatment of neurodegenerative diseases, have limited solubility in water. Their loading into PAMAM dendrimers will not only dramatically increase their solubility but will also allow them to be taken up via endocytosis to cross the BBB. This feature of PAMAM dendrimers is a major advantage over the use of proteins as nanocarriers, since proteins are highly compact nanostructures with negligible empty space in their interiors. Although the water solubility of the drug-encapsulated dendrimer may decrease compared to the free dendrimer, a major advantage of encapsulation (compared to surface attachment of drugs discussed below) is that the purity, size and surface of the dendrimer is unchanged; as previously mentioned, this is important for safety and predictable ADME profiles.

Among all the components of a PAMAM dendrimer, the surface has drawn the most attention. This is because macromolecules such as PAMAM dendrimers interact with cells and biomolecules predominantly via surface interactions. The surface therefore dictates the safety profile, which is one of the most important requirements for any therapeutic agent. Numerous studies have shown a strong correlation between the toxicity of PAMAM dendrimers and their surface functional groups. The amine-terminated dendrimer has been found to be the most toxic to cells compared to dendrimers with –OH or –COOH groups. This is because amines are positively charged under physiological conditions. Although amines are found on the surface of proteins, the toxicity of an amine-terminated dendrimer is due to the very high amounts of amines per given surface area (charge density), a property that results from its dendritic architecture. The high positive charge density of the amine-terminated dendrimer is responsible for its rapid and strong interaction with most biomolecules and cells (which have many negative charges on their surfaces), resulting in dendrimer toxicity [33]. For example, when mixed with red blood cells (RBCs) *in vitro*, these dendrimers form holes in the RBCs and cause hemolysis. When injected into the bloodstream, they would also readily attract complex acidic proteins (with isoelectric point (pI) values less than seven) that are abundantly present in the extracellular medium, resulting in altered ADME profiles. Several strategies have been used to alleviate this problem. Chemicals such as PEG and amino acids have been attached to the surface amines in order to reduce the charge density of the dendrimer. However, as noted above, such a strategy results in a mixture of products with different ADME profiles. In addition, altering the surface of the nanomolecule with PEG or amino acids will form unnatural surfaces that may be recognized by the immune system, resulting in the production of antibodies, loss of therapeutic efficacy and more importantly, adverse immune effects such as anaphylaxis [34]. On the other hand, parent PAMAM dendrimers with unmodified surfaces were found to be non-immunogenic [35]. The amines on the surface of the dendrimer are necessary to readily attach fluorescent dyes (or other imaging agents) for tracking and to complex and deliver nucleic acids for gene therapy, but are also toxic to cells. In order to solve this dilemma, an important question to answer is: how many amines are needed per dendrimer so that they can be safe but yet be functional? A major step towards this objective was recently reported by Srinageshwar and colleagues. They designed and synthesized a mixed-surface G4 PAMAM dendrimer composed of 58 hydroxyl groups (90%) and six amines (10%), known as the G4-90/10 dendrimer. In comparison, the parent G4 amine-terminated PAMAM dendrimer has 64 amine groups on its surface and no hydroxyl groups. These mixed-surface PAMAM dendrimers, which can be prepared by well-established dendrimer synthesis protocols (using excess reagents) at different hydroxyl/amine ratios, were found to be as pure as the parent dendrimers (as observed on electrophoresis gels and by RP-HPLC). In addition, the simple surface of the dendrimer, which is composed of mostly −OH and a few −NH_2_ groups, is probably responsible for its significantly improved safety profile. The G4-90/10 dendrimers were also found to cross the BBB when injected into the carotid artery of rodents [36]. Mixed-surface PAMAM dendrimers may therefore offer an excellent alternative to surface modified dendrimers as nanocarriers of drugs and genes for treating neurodegenerative diseases.

With these properties of PAMAM dendrimers in mind (especially safety as the top priority), let us look at the potential use of PAMAM dendrimers for the treatment of neurodegenerative diseases.

## 3. PAMAM Dendrimers and the Delivery of Small Molecule Drugs and Genes across the Blood Brain Barrier

The BBB refers to the selective membrane that forms the physical and chemical barriers between the blood and brain parenchyma. It has evolved to regulate the chemical environment of the central nervous system (CNS) through a variety of specialized cells and transporters. The BBB is composed of specialized capillary endothelial cells joined by tight junctions surrounded by extravascular components including the basal lamina, astrocyte foot processes, pericytes, and interneurons [39]. These components form a physical transport barrier that regulates the flow of nutrients in and waste out of the CNS. Additionally, a collection of efflux proteins and enzymes, including intracellular monoamine oxidase, cytochrome P450s, extracellular nucleases, and peptidases help make an active chemical barrier to protect the CNS from potentially toxic chemicals [40]. Notably, the endothelial cells of the BBB play a major role in regulating chemical homeostasis of the CNS and have unique properties compared to other systemic vascular endothelial cells. These cells contain a higher number of tight junctions (lack of fenestrations) which reduce the amount of paracellular movement of solutes and experience far lower rates of transcytosis compared to peripheral tissue, reducing vesicle-mediated transport of solutes [41].

The BBB does not completely halt the transport of substances into the brain. To function properly, the brain needs key nutrients, such as glucose, amino acids, nucleic acids, fatty acids, vitamins, and electrolytes, which are transported into the brain by numerous transporters and channels located on the endothelial membranes [42]. In addition to protein transporters, certain endogenous peptides and hormones, such as transferrin and insulin, may cross the BBB via receptor-mediated endocytosis [43]. Additionally, other types of BBB crossings exist, including: (1) small, water-soluble agents such as ions, which may cross by paracellular transport between tight junctions; (2) lipid-soluble chemicals, which may cross transcellularly, directly through the endothelial membrane; and (3) plasma proteins, which may cross via adsorptive endocytosis [40,44] or via receptor-mediated endocytosis. Examples of receptor-ligand interactions observed and used for receptor-mediated trafficking of nanomolecules across the BBB endothelial cells include transferrin [45], insulin [46], insulin-like growth factor II [47], low-density lipoprotein receptor-related protein (LRP) receptors [48], and diphtheria toxin receptors [49]. Since about 98% of small-molecule drugs fail to cross the BBB and few large-molecule drugs (e.g., insulin) cross, finding ways to deliver drugs across the BBB has been the focus of much research, which has, thus far, produced only mixed results [50].

How are PAMAM dendrimers used for delivery across the BBB? In order to answer this question, we must separate small molecule delivery (such as drugs < MW 500 Da) and macromolecule delivery (e.g., gene delivery MW > 500 Da). This is due to the significant differences in the size and surface characteristics of the products formed when dendrimers are loaded with drugs compared to dendrimers loaded with DNA or RNA (known as dendriplexes).

## 4. Delivery of Small Molecules

We shall discuss small molecule drugs first. These chemicals are either loaded into the cavities or attached to the surface of PAMAM dendrimers [51]. The size of the final dendrimer-drug product does not change appreciably (typically < 15 nm). Several chemotherapy agents have been loaded into PAMAM dendrimer cavities for treatment of a variety of cancers and diseases of other organs. However, surprisingly, there are hardly any reports about loading drugs within PAMAM dendrimer cavities for brain diseases. A few examples of this are given below:

- Carbamazepine (CBZ), an anti-epileptic drug, was shown to enhance autophagy and protect against neurodegeneration in vivo. However, it is poorly soluble in water and shows unpredictable pharmacokinetic profiles. Generation 4.5 carboxyl-terminated dendrimers were loaded with CBZ to yield stable, soluble and safe (as tested on RBCs and zebrafish) formulations for potential applications in the treatment of neurodegenerative diseases associated with toxicity of aggregated proteins such as Alzheimer’s disease, amyotrophic lateral sclerosis, Huntington’s disease and Parkinson’s disease [30]. No further studies were performed to see if the CBZ-dendrimer formulations were effective for the treatment of these diseases.
- He and co-workers encapsulated doxorubicin into G4 PAMAM dendrimers with surface PEG (PEGylated dendrimers) and the targeting ligands wheat germ agglutinin (WGA) and transferrin (Tf). The BBB-targeting ligands WGA and Tf increased the BBB permeability of the dendrimers. These nanoparticles were < 20 nm in size as measured by electron microscopy and dynamic light scattering. The PAMAM-PEG-WGA-Tf delivered more payload (doxorubicin) at brain tumor sites compared to free drug or dendrimers without Tf and WGA [52].
- Swami and colleagues loaded docetaxel (DTX) into G4 PAMAM dendrimers and covalently attached p-hydroxyl benzoic acid to the surface (pHBA). The pHBA has high affinity to sigma receptors that are predominant in the central nervous system. The G4-pHBA-DTX dendrimers were more effective in killing glioblastoma cells and delivered more DTX to the brain compared to free drug [53].

Compared to cavity loading, most researchers prefer covalent attachment of the drug to the surface of PAMAM dendrimers via hydrolysable amide or ester bonds. One reason for this may be due to the rapid release of encapsulated drugs compared to covalently linked drugs. For example, Patri and colleagues observed rapid release of PAMAM G5 encapsulated methotrexate in phosphate buffered saline compared to covalently coupled drug [54]. Clearly, a better way to encapsulate drugs within the dendrimer cavity is required to avoid changing the surface of the macromolecule. An example would be the entrapment of drugs within the dendritic branches of the dendrimers, which was demonstrated by Jansen and colleagues who introduced guest molecules during the construction phase of the dendrimer. This approach resulted in a significantly slower release of the guest molecules into solution, due to the dense packing of the dendrimer shell [55].

A few examples of the use of drugs covalently attached to PAMAM dendrimers for brain diseases are listed below:

- Microtubule inhibitors (estramustine and podophyllotoxin), covalently attached to PAMAM dendrimers, were found to be more effective in killing glioma cells compared to free drug [56].
- Teow and colleagues conjugated paclitaxel and lauryl chains on the surface of a G3 PAMAM dendrimer. The conjugates showed increased cytotoxicity and permeability across porcine brain endothelial cells [57].
- Sharma and colleagues found that minocycline, conjugated to G6 hydroxyl-terminated PAMAM dendrimers via amide linkages, reduced neuroinflammation in vivo when compared to free minocycline and did so at lower dosages, thus reducing potential drug toxicity [58].
- Kannan and colleagues conjugated *N*-acetyl-l-cysteine to a G4 hydroxylated PAMAM dendrimer via disulfide linkages, which could be cleaved by intracellular glutathione (GSH). This formulation was shown to reduce motor dysfunction in rabbit models with cerebral palsy when administered postnatally [59].
- Yang and Lopina showed that attaching venlafaxine, a SNRI antidepressant, to PAMAM dendrimer-PEG hydrogels via ester linkages provided an extended release formulation that may help patients with poor drug compliance [60].
- Gamage and colleagues showed that curcumin was conjugated onto a G3-succinamic acid surface dendrimer via ester bonds. This formulation was administered to rats implanted with human glioma cells. The G3-curcumin showed tumor specific distribution, suggesting a potential use for the treatment of brain cancer [61].

A combination of cavity loading and surface attachment has also been used. Li and co-workers prepared G4 PAMAM dendrimers with doxorubicin, PEG and transferrin attached to the surface while tamoxifen was encapsulated within the cavities of the dendrimer. The dendrimer conjugate crossed a BBB model via transferrin-mediated endocytosis and accumulated within glioma cells as tamoxifen-inhibited drug efflux proteins [62].

These studies on the use of PAMAM dendrimers for small molecule drug delivery, although still in its infancy, suggest the promise of PAMAM dendrimers for treating brain diseases. PAMAM dendrimers have also been used as drugs instead of carriers for drugs. The dendrimers by themselves decrease fibrillation of alpha-synuclein, with the anti-fibrillation effect increasing with the generation number and concentration of the dendrimer [63]. In addition, PAMAM dendrimers were found to promote the degradation of pre-existing alpha-synuclein fibers, providing potential benefit to Parkinson’s disease patients [64]. This anti-aggregation effect is also seen in Alzheimer’s disease models, where dendrimers have been shown to disrupt Aβ amyloid plaque formation and disruption of protease-resistant prion protein (PrPSc) in prion diseases, where protein aggregation is thought to play a key role in functional decline [65,66].

A summary of the various types of drugs delivered by PAMAM dendrimers for brain diseases is shown in Table 1.

## 5. Delivery of Genes

The interaction of dendrimers with nucleic acids is very different than small molecule drugs. Practically, only dendrimers with surface amines can be used for the delivery of nucleic acids (DNA or RNA). The amines form positive charges under physiological conditions that interact electrostatically with the negative charges on nucleic acids to form dendrimer-nucleic acid dendriplexes. Under physiological conditions (especially pH ~7), hydroxyls are neutral and carboxyls are anionic; thus, dendrimers with these surface groups will not bind and complex nucleic acids. Due to the multiple charges on both the amine-terminated dendrimers and nucleic acids, the dendriplexes formed can vary significantly in size and structure [69]. Several factors affect the nature of the dendriplexes including the size (generation) of the dendrimer, the size of the nucleic acid, the ratio of dendrimer amines (N) to nucleic acid phosphates (P) (also known as N/P or charge ratio), as well as solvent properties [70,71]. Dendrimer binding affinity to DNA increases with increasing generation. The complexes typically have low stability and tend to aggregate and precipitate at charge ratios close to one. In addition, higher dendrimer generations and charge ratios greater than 2 (twice as many amines as phosphates in the dendriplex) tend to form complexes with smaller size distributions and with better transfection efficiencies. In general, stable, uniform dendriplexes with nanometer dimensions are formed with higher generations and higher N/P ratios [69,71]. The transfection efficiency of dendrimer-nucleic acid complexes is difficult to predict, since it depends not only on the nature of the complexes formed, but also varies between different cell lines and even cell densities [70]. If preliminary biological results using dendriplexes are promising, it is advisable for researchers to carefully characterize the complexes formed by a variety of analytical techniques such as dynamic light scattering (DLS), agarose gel electrophoresis, fourier-transform infrared spectroscopy (FTIR), isothermal titration calorimetry (ITC), circular dichroism (CD )and differential scanning calorimetry (DSC) in order to ensure that similar complexes can be reproducibly prepared [69].

Several researchers have studied the delivery of genes with dendrimers. Excellent reviews exist on this subject [6,70]. Examples of PAMAM dendrimers used in gene delivery relevant to brain diseases are given below:

- One method of attenuating neurodegenerative disease progression is by slowing down the rate of neuronal death. This can be achieved by providing growth factors to the regions of neuronal degeneration, such as brain-derived neurotrophic factor (BDNF), glial-derived neurotropic factor (GDNF) and nerve growth factor (NGF) which are needed for the survival of particular neurons in the brain [72,73]. In HD, lack of BDNF results in the loss of medium spiny neurons of the striatum, a brain region involved in motor, cognitive, and emotional functions. As a result, HD patients present with deterioration in all three of these domains. Examples of this include chorea (an involuntary dance like movement), learning, and memory impairments as well as psychiatric problems. Since the lack of BDNF in the striatum results in the death of these cells, our laboratory has been involved in developing methods to increase BDNF production in the brains of HD rodent models. We have shown that increasing BDNF in HD mice (such as YAC128 and R6/2 models) resulted in the attenuation of motor and cognitive loss [73,74]. Shakhbazau and colleagues found that PAMAM G4 complexed with a plasmid for BDNF significantly increased the secretion of BDNF protein in human bone marrow mesenchymal stem cells (hMSCs), which can be implanted into diseased brains [75]. Although most researchers use charge ratios greater than one, it is interesting that these authors used a charge ratio of 1:1, giving a dendriplex size of about 150–200 nm.
- Similar complexes, formed between G4 and a plasmid for neurotrophin, were successfully used for transfecting human and rodent stem cells [75].

Unlike direct transfection of cells in culture, PAMAM-mediated delivery of genes in vivo often uses targeting ligands that are predominant in the brain. Examples of targeted delivery include:

- Huang and colleagues have shown that transferrin conjugated to PAMAM dendrimer-DNA dendriplexes increases gene expression approximately two-fold in the brain compared to dendriplexes that were not conjugated to transferrin [76].
- Additionally, the same researchers have shown in BALB/c mice that conjugating lactoferrin to PAMAM dendrimers with PEG spacers increased brain uptake of the dendrimer 4.6-fold, compared to non-conjugated PAMAM dendrimers and by a 2.2-fold increase compared to dendrimers conjugated to transferrin [77].
- LRP receptors have also been shown to be abundantly expressed in mammalian neuronal cells [78]. Angiopep has been used to target LRP receptors with some specificity [79]. Researchers have shown that gene expression was significantly increased in the cortex, caudate putamen, hippocampus and substantia nigra of BALB/c mice when Angiopep-PEG-PAMAM loaded with DNA was administered, as compared to unconjugated PAMAM loaded with DNA [80].
- Another receptor found on the BBB, the mannose 6-phosphate/insulin-like growth factor II receptor (M6P/IGFR2R), binds to M6P and IGFR2 at distinct sites. After the binding of M6P-tagged proteins, as well as IGF-2 on those receptors, the molecules may be internalized and sent to lysosomes for degradation [81]. Urayama and colleagues found that this receptor is highly expressed in neonatal mice compared to adult mice. Their findings showed increased uptake of radiolabeled β-glucuronidase in neonatal mice compared to adults, and that this uptake was inhibited by M6P via competitive inhibition [82]. Potentially, conjugating a dendrimer to one of the ligands of the M6P/IGFR2R could be therapeutic for neonatal diseases of the CNS.
- Another targeting ligand for CNS-enhanced drug delivery is the 29 amino-acid rabies virus glycoprotein (RVG29), which allows viral entry into the CNS by binding to nicotinic acetylcholine receptors on neurons [83]. Liu and colleagues have successfully exploited this interaction when RVG29 was conjugated to a PAMAM dendrimer loaded with DNA. They found that this conjugation crossed the BBB more efficiency *in vitro,* and had a preferential brain accumulation in vivo [84].
- Serramía and colleagues delivered a small siRNA systemically using carbosilane dendrimers targeting astrocytes in BALB/c mice and detected the presence of the dendrimers and the dendriplexes in the brain one hour and 24 hours following injection [85].

## 6. Fate of PAMAM Dendrimers in Cells

Crossing the blood brain barrier or brain cells requires that the drug-loaded dendrimer or the dendriplex attaches to cell membranes. If the dendrimer has a surface receptor, entry would be via receptor-mediated endocytosis. Otherwise, cells will use adsorptive endocytosis for uptake. The presence of a positive charge on the drug-loaded dendrimer or the dendriplex will enhance adsorptive endocytosis and subsequent cellular uptake. This is why dendrimers with positively charged surfaces readily cross the cell membrane and are more effective in delivering DNA/drugs to the cell in vitro and in vivo [33]. On the other hand, a completely neutral dendrimer having a 100% −OH surface will be less efficient to cross the hydrophobic cell membrane in order to deliver DNA/drugs [86]. This is also why dendriplexes with charge ratios greater than one are used for transfection.

The precise mechanism of how the dendrimer enters the cell, especially neurons and astrocytes, and how they release their drug and/or gene is not known. A few mechanisms for dendrimer uptake by cells have been proposed and include: (1) clathrin-mediated endocytosis; (2) caveolae-mediated endocytosis (specifically for G4 dendrimers); and (3) macropinocytosis [87]. Once inside the cytoplasm, the drug-loaded dendrimer or the dendriplex are enclosed within vesicles (endosomes). Normally, the endosomes mature as they become acidic and finally fuse with lysosomes. According to the proton sponge mechanism, it is believed that the acidification of the endosomes is suppressed, especially by the tertiary amines within the interior of the PAMAM dendrimer. This results in the entry of chloride ions and water into the endosomes (osmotic swelling). The swollen endosomes burst, releasing the dendrimer with its cargo. Under acidic conditions, most of the dendrimer tertiary amines become positively charged and repel the branches of the nanomolecule. This increases the dendrimer size and also helps in dislodging its bound drug or gene cargo [34,88].

## 7. Routes of Dendrimer Administration

The route of administration is also an important factor to consider when delivering dendrimers to the brain. The major challenge to brain delivery is to find an ideal route of administration that can maximize the effects of administered dendrimers on the CNS without causing systemic toxicity. Intravenous injection is the simplest method for achieving a rapid increase in blood concentration of the dendrimer and its payload. While directly injecting the dendrimer into the blood has the fastest pharmacokinetic properties, there are some undesirable aspects to this method. It has been shown by Kurokawa and colleagues that BBB penetration may not be effective. These authors found that intravenous administration of a G4 amine-terminated PAMAM dendrimer had poor CNS penetration due to their tendency to aggregate via non-Derjaguin-Landau-Verwey-Overbeek attractive forces originating from the surrounding divalent ions [88]. Neutral surface PAMAM dendrimers are better in this respect. Zhang and colleagues demonstrated that intravenous injection of G4-OH PAMAM dendrimers effectively penetrated CNS tumors in a rodent gliosarcoma model (with a compromised BBB). Additionally, they were able to reveal the homogeneous distribution of dendrimers throughout the solid tumor surrounding tissue fifteen minutes after injection. In a later study, Zhang and colleagues demonstrated in a canine model of neuro-inflammation that intravenously delivered G6-OH dendrimers showed extensive blood circulation and an extended half-life in cerebrospinal fluid (CSF) compared to the smaller G4-OH dendrimers administered intravenously. The authors also showed that brain penetration (concentration of drug in CSF/serum) correlated with the severity of neuro-inflammation and that the dendrimers preferentially targeted microglia and injured neuronal cells. Their research suggests that intravenous administration of larger dendrimers could provide extended therapy with significant CNS penetration [89]. Another way of increasing delivery to the brain is to attach a targeting ligand specific to, and/or abundantly found specifically on or abundant on brain cells. Unfortunately, no such ligand is available today that is unique to normal or diseased brain cells. Ligands such as transferrin [45], insulin [46], insulin-like growth factor II [47] and low-density lipoprotein receptor-related protein (LRP) receptors [48] are also found in other cells of the body.

Another way of enhancing the delivery of dendrimers to the brain is injection close to the brain via the carotid artery. Administration into the carotid artery increases the concentration of dendrimer reaching the brain. Srinageshwar and colleagues demonstrated that G4 PAMAM mixed-surface dendrimers composed of 90% −OH and 10% −NH_2_ surface molecules are taken up by neurons *in vitro*, cross the BBB following carotid artery administration, and integrate into neurons and glial cells of healthy mouse models with intact BBBs. No detectable amount of dendrimers were found in the peripheral organs (liver, lungs, and spleen) [35]. The small amount of positive charge on the G4-90/10 dendrimer (six amines/dendrimer) was also responsible for its improved safety profile (compared to an amine-terminated dendrimer, which has 64 amines/dendrimer) and allowed the dendrimer to stick to the endothelial cells for subsequent adsorptive endocytosis across the BBB. Although carotid injections are challenging and must be performed by highly trained professionals, this route of injection offers an excellent alternative for sending high amounts of drugs to the brain in case of life-threatening diseases such as glioblastoma multiforme.

Other than vascular injection, oral and intranasal dendrimer preparations have also been administered for brain uptake studies. Besides being the most common route of administration in the pharmaceutical market today, oral administration has the benefit of convenience and increased patient compliance. Challenges to oral delivery of all drugs has been intestinal epithelial penetration and first-pass metabolism in the liver [90]. In regard to the former barrier, dendrimers may actually increase intestinal absorption of therapeutics. In a previous study, it was found that doxorubicin levels in serum were 200-fold higher when orally administered with PAMAM dendrimers compared to free doxorubicin [91]. It was also found that there was a significantly higher transport of doxorubicin when using a doxorubicin–PAMAM complex compared to free doxorubicin in Caco-2 monolayers [92]. Studies with everted rat intestinal sac systems also showed high transport of PAMAM dendrimers, especially those with anionic surfaces [93], while other studies found less promising results using isolated human intestinal epithelium [94]. Although there is much work to be done in this area, oral administration of dendrimer-drug formulations could potentially provide an efficacious and convenient way of delivering drugs to the CNS. If the targeting ligand (mostly proteins) can withstand destruction in the gastrointestinal system, then oral formulations of dendrimers conjugated to a BBB-targeting moiety may help enhance delivery to the brain [91].

Perhaps more promising than oral formulations, intranasal preparations of dendrimers offer the benefits of being non-invasive without the same metabolic issues of oral drugs [95]. Win-Shwe and colleagues found that a single dose of PAMAM dendrimers intranasally administered to eight-week-old BALB/c mice upregulated BDNF mRNA in the hippocampus and cerebral cortex of treated mice. This study suggests that the dendrimers entered the CNS via systemic circulation or through olfactory nerve routes to alter gene expression, however no analyses were done to confirm the presence of dendrimers in the affected brain areas [96]. Katare and colleagues took this approach one step further and complexed water-insoluble haloperidol with a dendrimer formulation and found a 100-fold increase in aqueous solubility. The dendrimer formulation was seven times more efficient at targeting haloperidol to the striatum when administered intranasally, compared to intraperitoneal injection. Additionally, an intranasal dose 6.7 times lower than the intraperitoneal dose produced comparable cataleptic and locomotor behavioral responses. However, while a 6.7 times lower dose produced similar responses, intranasal administration produced a haloperidol concentration two to three times lower in the striatum compared to intraperitoneal injection without dendrimers [67]. While the striatal concentrations were lower, an increased efficiency and lower dose that produce the intended response should not be discounted.

## 8. Conclusions

Keeping safety and reproducibility in preparation of dendrimer-drug formulations as the top two priorities for the potential use of these nanomolecules for treating brain diseases, PAMAM dendrimers with −OH surfaces are the preferred choice since they do not stick to surfaces, diffuse well throughout the brain, are non-immunogenic and are highly water-soluble. Although cellular uptake is better for amine-surface dendrimers, the compromised BBB associated with the diseased state can facilitate targeting the hydroxyl surface PAMAM dendrimers to the affected sites of the brain. The use of mixed-surface dendrimers with limited amines on the dendrimer surface, such as the G4 90/10, is another option that provides safety, the ability to bind drugs and DNA, improved BBB penetration and cellular uptake. It is also advisable to limit surface changes to the dendrimer to maintain its benefits, such as purity and safety, and avoid unpredictable ADME profiles. For this reason, dendrimer cavities should be used for loading drugs by encapsulation rather than by attaching them to the surface. The rapid release of encapsulated drugs in vivo can be solved by entrapment, which results in a much slower release of the payload. In the case of gene delivery, where amines are required for DNA binding and compaction, use of mixed-surface dendrimers that have the minimal amines required to carry the gene and with the dendrimer surface decorated with the more biocompatible hydroxyl groups should be considered. If safe and high purity dendrimer formulations are used, high doses may be used to deliver sufficient drug or gene to the brain. The compromised BBB associated with the diseased state can facilitate targeting the payload to only the affected sites of the brain.

## Figures and Tables

**Table 1:** Applications of PAMAM Dendrimers for Brain Disease.

Drug(s)	Loading Method	Application	Results
Carbamazepine	Encapsulation within a G4.5 carboxyl-terminated dendrimer	Neurodegenerative diseases	Decreased neurodegeneration in vivo, decreased protein aggregation, enhanced autophagy, and increased drug solubility [30]
Curcumin	Covalent linkage to a G3-succinamic acid surface dendrimer via ester bonds	Glioma	Increased delivery in a tumor-specific distribution [61]
Docetaxel	Encapsulation within G4 PAMAM dendrimers with covalently attached pHBA	Glioblastoma	Increased glioblastoma-cell death, and increased drug delivery to the brain [53]
Doxorubicin	Encapsulation within PEGylated G4 PAMAM dendrimers with WGA and Tf targeting ligands	Brain tumors	Increased doxorubicin payload at tumor sites [52]
Estramustine and podophyllotoxin	Covalent linkage to PAMAM dendrimers	Glioma	More effective killing of glioma cells [56]
Haloperidol	Encapsulation within a G5 PAMAM dendrimer with 1,4-diaminobutane core	Psychiatric	Increased brain and plasma concentrations of haloperidol compared to control formulation in a rat model [67]
Minocycline	Covalent linkage to G6 hydroxyl-terminated PAMAM dendrimers via amide linkages	Stroke	Reduced neuroinflammation in vivo at lower doses [58]
*N*-acetyl-l-cysteine	Covalent linkage to a G4 hydroxylated PAMAM dendrimer via disulfide linkages	Cerebral palsy	Reduced motor dysfunction in rabbit models [59]
Paclitaxel	Covalent linkage to G3 PAMAM dendrimers with added lauryl chains	Brain tumors	Increased cytotoxicity and permeability across porcine brain endothelial cells [57]
Risperidone	Encapsulation within a G4 PAMAM dendrimer	Psychiatric	Increased aqueous solubility of risperidone without significant hemolysis or morphological changes to human red blood cells [68]
Tamoxifen and doxorubicin	Combination encapsulation (tamoxifen) and covalent linkage (doxorubicin) to G4 PAMAM dendrimers with added PEG and Tf	Glioma	Increased accumulation within glioma cells [62]
Venlafaxine	Covalent linkage to PAMAM dendrimers-PEG hydrogels via ester linkages	Psychiatric	Extended release [60]

PAMAM—polyamidoamine; PEG—polyethylene glycol; pHBA—p-hydroxyl benzoic acid; Tf—transferrin; WGA—wheat germ antigen.

## References

[1] Huebner E.A., Strittmatter S.M. (2009). Axon regeneration in the peripheral and central nervous systems. Results Probl. Cell Differ..

[2] Young A.B. (2009). Four decades of neurodegenerative disease research: How far we have come!. J. Neurosci..

[3] Holland E.C. (2000). Glioblastoma multiforme: The terminator. Proc. Natl. Acad. Sci. USA.

[4] Kannan R.M., Nance E., Kannan S., Tomalia D.A. (2014). Emerging concepts in dendrimer-based nanomedicine: From design principles to clinical applications. J. Intern. Med..

[5] Otto D.P., de Villiers M.M. (2018). Poly(amidoamine) Dendrimers as a Pharmaceutical Excipient. Are We There yet?. J. Pharm. Sci..

[6] Palmerston Mendes L., Pan J., Torchilin V.P. (2017). Dendrimers as Nanocarriers for Nucleic Acid and Drug Delivery in Cancer Therapy. Mol. Basel Switz..

[7] Menjoge A.R., Kannan R.M., Tomalia D.A. (2010). Dendrimer-based drug and imaging conjugates: Design considerations for nanomedical applications. Drug Discov. Today.

[8] Esfand R., Tomalia D.A. (2001). Poly(amidoamine) (PAMAM) dendrimers: From biomimicry to drug delivery and biomedical applications. Drug Discov. Today.

[9] Zhang F., Lin Y.-A., Kannan S., Kannan R.M. (2016). Targeting specific cells in the brain with nanomedicines for CNS therapies. J. Control. Release.

[10] Somani S., Dufès C. (2014). Applications of dendrimers for brain delivery and cancer therapy. Nanomedicine.

[11] Boyd B.J., Kaminskas L.M., Karellas P., Krippner G., Lessene R., Porter C.J.H. (2006). Cationic poly-l-lysine dendrimers: Pharmacokinetics, biodistribution, and evidence for metabolism and bioresorption after intravenous administration to rats. Mol. Pharm..

[12] Kazmierczak-Baranska J., Pietkiewicz A., Janicka M., Wei Y., Turrin C.-O., Majoral J.-P., Nawrot B., Caminade A.-M. (2010). Synthesis of a fluorescent cationic phosphorus dendrimer and preliminary biological studies of its interaction with DNA. Nucleosides Nucleotides Nucleic Acids.

[13] Wrobel D., Kubikova R., Müllerová M., Strašák T., RůŽička K., Fulem M., Maly J. (2018). Phosphonium carbosilane dendrimers—Interaction with a simple biological membrane model. Phys. Chem. Chem. Phys..

[14] Abbasi E., Aval S.F., Akbarzadeh A., Milani M., Nasrabadi H.T., Joo S.W., Hanifehpour Y., Nejati-Koshki K., Pashaei-Asl R. (2014). Dendrimers: Synthesis, applications, and properties. Nanoscale Res. Lett..

[15] Nwe K., Milenic D.E., Ray G.L., Kim Y.-S., Brechbiel M.W. (2012). Preparation of cystamine core dendrimer and antibody-dendrimer conjugates for MRI angiography. Mol. Pharm..

[16] Caminade A.-M., Laurent R., Majoral J.-P. (2005). Characterization of dendrimers. Adv. Drug Deliv. Rev..

[17] Dendrimers and Other Dendritic Polymers. https://www.wiley.com/en-us/Dendrimers+and+Other+Dendritic+Polymers-p-9780471638506.

[18] Dougherty C.A., Vaidyanathan S., Orr B.G., Banaszak Holl M.M. (2015). Fluorophore:dendrimer ratio impacts cellular uptake and intracellular fluorescence lifetime. Bioconjug. Chem..

[19] Albertazzi L., Gherardini L., Brondi M., Sulis Sato S., Bifone A., Pizzorusso T., Ratto G.M., Bardi G. (2013). In vivo distribution and toxicity of PAMAM dendrimers in the central nervous system depend on their surface chemistry. Mol. Pharm..

[20] Yu M., Zheng J. (2015). Clearance Pathways and Tumor Targeting of Imaging Nanoparticles. ACS Nano.

[21] Sharma A. (2016). Medical Biochemistry: Molecules to Disease, Understanding & Applying Biochemistry in Healthcare.

[22] Lesniak W.G., Mishra M.K., Jyoti A., Balakrishnan B., Zhang F., Nance E., Romero R., Kannan S., Kannan R.M. (2013). Biodistribution of fluorescently labeled PAMAM dendrimers in neonatal rabbits: Effect of neuroinflammation. Mol. Pharm..

[23] Mishra M.K., Beaty C.A., Lesniak W.G., Kambhampati S.P., Zhang F., Wilson M.A., Blue M.E., Troncoso J.C., Kannan S., Johnston M.V. (2014). Dendrimer brain uptake and targeted therapy for brain injury in a large animal model of hypothermic circulatory arrest. ACS Nano.

[24] Posadas I., Monteagudo S., Ceña V. (2016). Nanoparticles for brain-specific drug and genetic material delivery, imaging and diagnosis. Nanomedicine.

[25] Sarin H., Kanevsky A.S., Wu H., Brimacombe K.R., Fung S.H., Sousa A.A., Auh S., Wilson C.M., Sharma K., Aronova M.A. (2008). Effective transvascular delivery of nanoparticles across the blood-brain tumor barrier into malignant glioma cells. J. Transl. Med..

[26] Nance E., Zhang F., Mishra M.K., Zhang Z., Kambhampati S.P., Kannan R.M., Kannan S. (2016). Nanoscale effects in dendrimer-mediated targeting of neuroinflammation. Biomaterials.

[27] Park E.J., Cho H., Kim S.W., Na D.H. (2014). Chromatographic methods for characterization of poly(ethylene glycol)-modified polyamidoamine dendrimers. Anal. Biochem..

[28] Park E.J., Na D.H. (2015). Difference in microchip electrophoretic mobility between partially and fully PEGylated poly(amidoamine) dendrimers. Anal. Biochem..

[29] Sharma A., Desai A., Ali R., Tomalia D. (2005). Polyacrylamide gel electrophoresis separation and detection of polyamidoamine dendrimers possessing various cores and terminal groups. J. Chromatogr. A.

[30] Igartúa D.E., Martinez C.S., Temprana C.F., Alonso S.D.V., Prieto M.J. (2018). PAMAM dendrimers as a carbamazepine delivery system for neurodegenerative diseases: A biophysical and nanotoxicological characterization. Int. J. Pharm..

[31] Choudhary S., Gupta L., Rani S., Dave K., Gupta U. (2017). Impact of Dendrimers on Solubility of Hydrophobic Drug Molecules. Front. Pharmacol..

[32] Arima H., Motoyama K., Higashi T. (2014). Cyclodextrin/Dendrimer conjugates as DNA and oligonucleotide carriers. Curr. Top. Med. Chem..

[33] Vidal F., Guzman L. (2015). Dendrimer nanocarriers drug action: Perspective for neuronal pharmacology. Neural Regen. Res..

[34] Zhang P., Sun F., Liu S., Jiang S. (2016). Anti-PEG antibodies in the clinic: Current issues and beyond PEGylation. J. Control. Release.

[35] Roberts J.C., Bhalgat M.K., Zera R.T. (1996). Preliminary biological evaluation of polyamidoamine (PAMAM) Starburst dendrimers. J. Biomed. Mater. Res..

[36] Srinageshwar B., Peruzzaro S., Andrews M., Johnson K., Hietpas A., Clark B., McGuire C., Petersen E., Kippe J., Stewart A. (2017). PAMAM Dendrimers Cross the Blood–Brain Barrier When Administered through the Carotid Artery in C57BL/6J Mice. Int. J. Mol. Sci..

[37] Kesharwani P., Banerjee S., Gupta U., Mohd Amin M.C.I., Padhye S., Sarkar F.H., Iyer A.K. (2015). PAMAM dendrimers as promising nanocarriers for RNAi therapeutics. Mater. Today.

[38] Arseneault M., Wafer C., Morin J.-F. (2015). Recent advances in click chemistry applied to dendrimer synthesis. Mol. Basel Switz..

[39] Xu L., Zhang H., Wu Y. (2013). Dendrimer Advances for the Central Nervous System Delivery of Therapeutics. ACS Chem. Neurosci..

[40] Abbott N.J., Patabendige A.A.K., Dolman D.E.M., Yusof S.R., Begley D.J. (2010). Structure and function of the blood-brain barrier. Neurobiol. Dis..

[41] Coomber B.L., Stewart P.A. (1985). Morphometric analysis of CNS microvascular endothelium. Microvasc. Res..

[42] Simpson I.A., Carruthers A., Vannucci S.J. (2007). Supply and demand in cerebral energy metabolism: The role of nutrient transporters. J. Cereb. Blood Flow Metab..

[43] Lajoie J.M., Shusta E.V. (2015). Targeting receptor-mediated transport for delivery of biologics across the blood-brain barrier. Annu. Rev. Pharmacol. Toxicol..

[44] Pardridge W.M. (2003). Blood-brain barrier drug targeting: The future of brain drug development. Mol. Interv..

[45] Hervé F., Ghinea N., Scherrmann J.-M. (2008). CNS delivery via adsorptive transcytosis. AAPS J..

[46] Wu D., Yang J., Pardridge W.M. (1997). Drug targeting of a peptide radiopharmaceutical through the primate blood-brain barrier in vivo with a monoclonal antibody to the human insulin receptor. J. Clin. Investig..

[47] Reinhardt R.R., Bondy C.A. (1994). Insulin-like growth factors cross the blood-brain barrier. Endocrinology.

[48] Kreuter J., Ramge P., Petrov V., Hamm S., Gelperina S.E., Engelhardt B., Alyautdin R., von Briesen H., Begley D.J. (2003). Direct evidence that polysorbate-80-coated poly(butylcyanoacrylate) nanoparticles deliver drugs to the CNS via specific mechanisms requiring prior binding of drug to the nanoparticles. Pharm. Res..

[49] Wang P., Xue Y., Shang X., Liu Y. (2010). Diphtheria toxin mutant CRM197-mediated transcytosis across blood-brain barrier in vitro. Cell. Mol. Neurobiol..

[50] Pardridge W.M. (2002). Drug and gene delivery to the brain: The vascular route. Neuron.

[51] Kim Y., Park E.J., Na D.H. (2018). Recent progress in dendrimer-based nanomedicine development. Arch. Pharm. Res..

[52] He H., Li Y., Jia X.-R., Du J., Ying X., Lu W.-L., Lou J.-N., Wei Y. (2011). PEGylated Poly(amidoamine) dendrimer-based dual-targeting carrier for treating brain tumors. Biomaterials.

[53] Swami R., Singh I., Kulhari H., Jeengar M.K., Khan W., Sistla R. (2017). Correction to: p-Hydroxy benzoic acid-conjugated dendrimer nanotherapeutics as potential carriers for targeted drug delivery to brain: An in vitro and in vivo evaluation. J. Nanopart. Res..

[54] Patri A.K., Kukowska-Latallo J.F., Baker J.R. (2005). Targeted drug delivery with dendrimers: Comparison of the release kinetics of covalently conjugated drug and non-covalent drug inclusion complex. Adv. Drug Deliv. Rev..

[55] Jansen J.F., de Brabander-van den Berg E.M., Meijer E.W. (1994). Encapsulation of guest molecules into a dendritic box. Science.

[56] Sk U.H., Dixit D., Sen E. (2013). Comparative study of microtubule inhibitors—Estramustine and natural podophyllotoxin conjugated PAMAM dendrimer on glioma cell proliferation. Eur. J. Med. Chem..

[57] Teow H.M., Zhou Z., Najlah M., Yusof S.R., Abbott N.J., D’Emanuele A. (2013). Delivery of paclitaxel across cellular barriers using a dendrimer-based nanocarrier. Int. J. Pharm..

[58] Sharma R., Kim S.-Y., Sharma A., Zhang Z., Kambhampati S.P., Kannan S., Kannan R.M. (2017). Activated Microglia Targeting Dendrimer-Minocycline Conjugate as Therapeutics for Neuroinflammation. Bioconjug. Chem..

[59] Kannan S., Dai H., Navath R.S., Balakrishnan B., Jyoti A., Janisse J., Romero R., Kannan R.M. (2012). Dendrimer-based postnatal therapy for neuroinflammation and cerebral palsy in a rabbit model. Sci. Transl. Med..

[60] Yang H., Lopina S.T. (2005). Extended release of a novel antidepressant, venlafaxine, based on anionic polyamidoamine dendrimers and poly(ethylene glycol)-containing semi-interpenetrating networks. J. Biomed. Mater. Res. A.

[61] Gamage N., Jing L., Worsham M., Ali M. (2016). Targeted Theranostic Approach for Glioma Using Dendrimer-Based Curcumin Nanoparticle. J. Nanomed. Nanotechnol..

[62] Li Y., He H., Jia X., Lu W.-L., Lou J., Wei Y. (2012). A dual-targeting nanocarrier based on poly(amidoamine) dendrimers conjugated with transferrin and tamoxifen for treating brain gliomas. Biomaterials.

[63] Milowska K., Malachowska M., Gabryelak T. (2011). PAMAM G4 dendrimers affect the aggregation of α-synuclein. Int. J. Biol. Macromol..

[64] Rekas A., Lo V., Gadd G.E., Cappai R., Yun S.I. (2009). PAMAM dendrimers as potential agents against fibrillation of alpha-synuclein, a Parkinson’s disease-related protein. Macromol. Biosci..

[65] Klajnert B., Cladera J., Bryszewska M. (2006). Molecular interactions of dendrimers with amyloid peptides: pH dependence. Biomacromolecules.

[66] Cordes H., Boas U., Olsen P., Heegaard P.M.H. (2007). Guanidino- and urea-modified dendrimers as potent solubilizers of misfolded prion protein aggregates under non-cytotoxic conditions. dependence on dendrimer generation and surface charge. Biomacromolecules.

[67] Katare Y.K., Daya R.P., Sookram Gray C., Luckham R.E., Bhandari J., Chauhan A.S., Mishra R.K. (2015). Brain Targeting of a Water Insoluble Antipsychotic Drug Haloperidol via the Intranasal Route Using PAMAM Dendrimer. Mol. Pharm..

[68] Prieto M.J., Temprana C.F., del Río Zabala N.E., Marotta C.H., del Valle Alonso S. (2011). Optimization and in vitro toxicity evaluation of G4 PAMAM dendrimer-risperidone complexes. Eur. J. Med. Chem..

[69] Braun C.S., Vetro J.A., Tomalia D.A., Koe G.S., Koe J.G., Middaugh C.R. (2005). Structure/function relationships of polyamidoamine/DNA dendrimers as gene delivery vehicles. J. Pharm. Sci..

[70] Dufès C., Uchegbu I.F., Schätzlein A.G. (2005). Dendrimers in gene delivery. Adv. Drug Deliv. Rev..

[71] Shen X.-C., Zhou J., Liu X., Wu J., Qu F., Zhang Z.-L., Pang D.-W., Quéléver G., Zhang C.-C., Peng L. (2007). Importance of size-to-charge ratio in construction of stable and uniform nanoscale RNA/dendrimer complexes. Org. Biomol. Chem..

[72] Deng X., Liang Y., Lu H., Yang Z., Liu R., Wang J., Song X., Long J., Li Y., Lei D. (2013). Co-transplantation of GDNF-overexpressing neural stem cells and fetal dopaminergic neurons mitigates motor symptoms in a rat model of Parkinson’s disease. PLoS ONE.

[73] Dey N.D., Bombard M.C., Roland B.P., Davidson S., Lu M., Rossignol J., Sandstrom M.I., Skeel R.L., Lescaudron L., Dunbar G.L. (2010). Genetically engineered mesenchymal stem cells reduce behavioral deficits in the YAC 128 mouse model of Huntington’s disease. Behav. Brain Res..

[74] Rossignol J., Fink K.D., Crane A.T., Davis K.K., Bombard M.C., Clerc S., Bavar A.M., Lowrance S.A., Song C., Witte S. (2015). Reductions in behavioral deficits and neuropathology in the R6/2 mouse model of Huntington’s disease following transplantation of bone-marrow-derived mesenchymal stem cells is dependent on passage number. Stem Cell Res. Ther..

[75] Shakhbazau A., Shcharbin D., Seviaryn I., Goncharova N., Kosmacheva S., Potapnev M., Gabara B., Ionov M., Bryszewska M. (2010). Use of polyamidoamine dendrimers to engineer BDNF-producing human mesenchymal stem cells. Mol. Biol. Rep..

[76] Huang R.-Q., Qu Y.-H., Ke W.-L., Zhu J.-H., Pei Y.-Y., Jiang C. (2007). Efficient gene delivery targeted to the brain using a transferrin-conjugated polyethyleneglycol-modified polyamidoamine dendrimer. FASEB J..

[77] Huang R., Ke W., Liu Y., Jiang C., Pei Y. (2008). The use of lactoferrin as a ligand for targeting the polyamidoamine-based gene delivery system to the brain. Biomaterials.

[78] Bu G., Maksymovitch E.A., Nerbonne J.M., Schwartz A.L. (1994). Expression and function of the low density lipoprotein receptor-related protein (LRP) in mammalian central neurons. J. Biol. Chem..

[79] Demeule M., Currie J.-C., Bertrand Y., Ché C., Nguyen T., Régina A., Gabathuler R., Castaigne J.-P., Béliveau R. (2008). Involvement of the low-density lipoprotein receptor-related protein in the transcytosis of the brain delivery vector angiopep-2. J. Neurochem..

[80] Ke W., Shao K., Huang R., Han L., Liu Y., Li J., Kuang Y., Ye L., Lou J., Jiang C. (2009). Gene delivery targeted to the brain using an Angiopep-conjugated polyethyleneglycol-modified polyamidoamine dendrimer. Biomaterials.

[81] Auletta M., Nielsen F.C., Gammeltoft S. (1992). Receptor-mediated endocytosis and degradation of insulin-like growth factor I and II in neonatal rat astrocytes. J. Neurosci. Res..

[82] Urayama A., Grubb J.H., Sly W.S., Banks W.A. (2004). Developmentally regulated mannose 6-phosphate receptor-mediated transport of a lysosomal enzyme across the blood-brain barrier. Proc. Natl. Acad. Sci. USA.

[83] Kumar P., Wu H., McBride J.L., Jung K.-E., Kim M.H., Davidson B.L., Lee S.K., Shankar P., Manjunath N. (2007). Transvascular delivery of small interfering RNA to the central nervous system. Nature.

[84] Liu Y., Bryantsev V.S., Diallo M.S., Goddard W.A. (2009). PAMAM dendrimers undergo pH responsive conformational changes without swelling. J. Am. Chem. Soc..

[85] Serramía M.J., Álvarez S., Fuentes-Paniagua E., Clemente M.I., Sánchez-Nieves J., Gómez R., de la Mata J., Muñoz-Fernández M.Á. (2015). In vivo delivery of siRNA to the brain by carbosilane dendrimer. J. Control. Release.

[86] Márquez-Miranda V., Peñaloza J.P., Araya-Durán I., Reyes R., Vidaurre S., Romero V., Fuentes J., Céric F., Velásquez L., González-Nilo F.D., Otero C. (2016). Effect of Terminal Groups of Dendrimers in the Complexation with Antisense Oligonucleotides and Cell Uptake. Nanoscale Res. Lett..

[87] Vidal F., Vásquez P., Díaz C., Nova D., Alderete J., Guzmán L. (2016). Mechanism of PAMAM Dendrimers Internalization in Hippocampal Neurons. Mol. Pharm..

[88] Kurokawa Y., Sone H., Win-Shwe T.-T., Zeng Y., Kimura H., Koyama Y., Yagi Y., Matsui Y., Yamazaki M., Hirano S. (2017). Aggregation is a critical cause of poor transfer into the brain tissue of intravenously administered cationic PAMAM dendrimer nanoparticles. Int. J. Nanomed..

[89] Zhang F., Trent Magruder J., Lin Y.-A., Crawford T.C., Grimm J.C., Sciortino C.M., Wilson M.A., Blue M.E., Kannan S., Johnston M.V. (2017). Generation-6 hydroxyl PAMAM dendrimers improve CNS penetration from intravenous administration in a large animal brain injury model. J. Control. Release.

[90] Thanki K., Gangwal R.P., Sangamwar A.T., Jain S. (2013). Oral delivery of anticancer drugs: Challenges and opportunities. J. Control. Release.

[91] Ke W., Zhao Y., Huang R., Jiang C., Pei Y. (2008). Enhanced oral bioavailability of doxorubicin in a dendrimer drug delivery system. J. Pharm. Sci..

[92] Sadekar S., Ghandehari H. (2012). Transepithelial transport and toxicity of PAMAM dendrimers: Implications for oral drug delivery. Adv. Drug Deliv. Rev..

[93] Wiwattanapatapee R., Carreño-Gómez B., Malik N., Duncan R. (2000). Anionic PAMAM dendrimers rapidly cross adult rat intestine in vitro: A potential oral delivery system?. Pharm. Res..

[94] Hubbard D., Enda M., Bond T., Moghaddam S.P.H., Conarton J., Scaife C., Volckmann E., Ghandehari H. (2015). Transepithelial Transport of PAMAM Dendrimers Across Isolated Human Intestinal Tissue. Mol. Pharm..

[95] Lu C.-T., Zhao Y.-Z., Wong H.L., Cai J., Peng L., Tian X.-Q. (2014). Current approaches to enhance CNS delivery of drugs across the brain barriers. Int. J. Nanomed..

[96] Win-Shwe T.-T., Sone H., Kurokawa Y., Zeng Y., Zeng Q., Nitta H., Hirano S. (2014). Effects of PAMAM dendrimers in the mouse brain after a single intranasal instillation. Toxicol. Lett..

